# Is There Safety Outside the “Safe Zone” in Total Hip Replacement? A Retrospective Observational Study

**DOI:** 10.3390/medicina60050772

**Published:** 2024-05-07

**Authors:** Piotr Stępiński, Dawid Jegierski, Grzegorz Maciąg, Bartosz Maciąg, Olga Adamska, Artur Stolarczyk

**Affiliations:** 1 Clinic for Orthopedics and Rehabilitation, Medical University of Warsaw, 04-749 Warsaw, Poland; ortopedia@mssw.pl (D.J.); bartosz.maciag94@gmail.com (B.M.); drstolarczyk@gmail.com (A.S.); 2 Faculty of Medicine, Medical University of Warsaw, 02-091 Warsaw, Poland; g.maciag.14@gmail.com (G.M.); olgaadam98@gmail.com (O.A.)

**Keywords:** total hip replacement, arthroplasty, anteversion, spinopelvic, THA, THR

## Abstract

*Background and Objectives*: The safe zone in total hip replacement was introduced many years ago. Its aim was to provide guidelines for orthopedic surgeons in order to avoid complications such as instability. With the growing interest in spinopelvic alignment, some new insights suggest that the safe zone is an obsolete concept. This study aims to show that, even outside the safe zone, the effect of total hip replacement can be satisfactory. This could be used as preliminary study for an analysis of a larger group. *Materials and Methods*: Fifty-nine consecutive patients with end-stage osteoarthritis treated by total hip replacement were enrolled into the study and divided into two groups: inside the safe zone and outside the safe zone. A physical examination during postoperative visits was performed; the range of movement was measured using a goniometer; and the HHS and VAS were taken to measure functional outcomes and pain, respectively. An analysis of the radiological outcomes was performed. *Results:* There was no significant difference in regard to changes in total offset, pain, HHS and other complications. There were no signs of instability among patients during the follow-up. *Conclusions*: The results of this study show that the “safe zone” is a more complicated term that was previously thought. A proper soft tissue balance and spinopelvic alignment could be factors that change the “safe zone” for each patient and make it more individual.

## 1. Introduction

Total hip replacement (THA) is one of the most successful operations of all time, and, due to this, it is also one of the most commonly performed nowadays in orthopedic surgery. With a survival rate of 95% for 10 years and over 1 million operations performed worldwide each year [1], it leads to a huge number of patients and potential problems. In 2019, these numbers significantly decreased due to the COIVD-19 pandemic, to about 69% of those of the previous year [2]. Now, a significant increase can be seen. Thus, the demand for new knowledge and updates regarding this topic is significant [3]. Especially useful are data that may have an impact on preoperative planning, which could lead to a better understanding of implant placement and its consequences, to prevent mistakes in the future [4]. In recent years, many research advancements have considerably improved this process—for example, using femoral heads with large diameters was proven to give better results [5], and taking into account spinopelvic alignment is now a matter of high importance to predict results [6], prevent complications [7] or place the cup correctly [8].

The acetabular cup position is best described by offset, anteversion and inclination, which are crucial parameters in operative planning. There are three types of offset: femoral, which is described as the shortest distance between the center of rotation and the femoral axis; acetabular, which is described as the shortest distance from the pubic symphysis (midline) to the center of rotation; and global, which is the sum of the femoral and acetabular values. Anteversion is defined as the angle between a line connecting the lateral anterior and posterior margins of the acetabular component and the sagittal plane, and inclination is described as the angle between the longitudinal axis of the body and the projection of the acetabular axis in the coronal plane.

The proper positioning of the acetabular component of the endoprosthesis is important in minimizing the risk of complications due to the operation, such as hip instability, improper polyethylene wear or iliopsoas impingement [9]. There are many factors that may influence the operation, such as pre-existing deformities, poor bone quality, the surgical technique and implant selection. Since patients’ factors are difficult to change, there is always a need for significant improvements in other fields, such as the design of implants, better surgical techniques, etc. Clinicians also devote effort to the proper preoperative planning to avoid complications, although there is always a possibility of underestimating some factor that is crucial. A few of these factors have already been identified as important and proven to affect the cup placement, such as obesity [10], the handedness of the surgeon [11] and the axial rotation of the patient on the operating table [12]. Thus far, the impact of the offset on the abductor lever arm, instability and function has been well described [4,10]. Proper soft tissue tensioning achieved by offset restoration has a positive influence on abductor muscle function and hip stability; excessive soft tissue tension may lead to faster polyethylene wear; and decreased soft tissue tension may lead to instability and improper muscle function, which sometimes results in the Trendelenburg sign. Besides the offset, the angular position is another vital parameter; in particular, anteversion is believed to have an important role in the stability and function of the hip [4,10] and is believed to be still underinvestigated. Suboptimal angular positioning may pose a huge problem for patients in their daily living activities and lead to lower satisfaction following treatment, and it may sometimes pose a major challenge for surgeons [13].

Problems with the proper angular placement of the cup have been examined for many years. Regarding modern arthroplasty, there has been a debate about the perfect anteversion and inclination of this part of the implant. It is well known that the biomechanics after total hip replacement change. The results of some studies suggest that patients with THA have dysfunction of the stabilizer muscles of the lumbopelvic region, along with increased pelvic motion [14]. These findings should be taken into account during preoperative planning, because any dysfunction in the stabilizer muscles can lead to instability, which is a serious complication.

**Figure 1:**
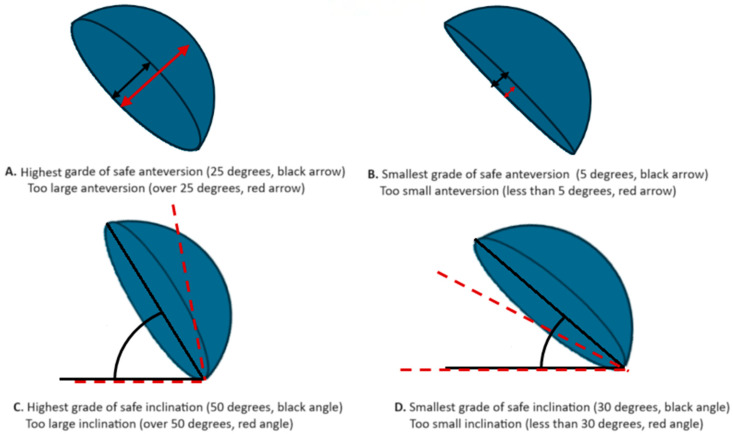
Visualization of Lewinnek’s safe zone concept.

Over 40 years ago, Lewinnek investigated the problem of stability and established the “safe zone” (anteversion 15 degrees ± 10 and inclination degrees 40 ± 10) according to research performed on patients with postoperative hip instability [15]. This approach to cup placement has been used as a guideline for orthopedic surgeons for decades. The visualization of this concept is presented in Figure 1. Unfortunately, it has been shown that the cup anteversion range widely considered as “safe” does not provide adequate stability in every case, suggesting that there is a “functional safe zone” that is more important for stability [16]. Although more difficult to implement in everyday operative planning, this new approach is more individual and potentially can lead to a decrease in complications. These new ideas have led to further research [13] and increased interest in this area, which is thought to be critical in achieving better postoperative results.

Almost every surgical intervention in the musculoskeletal system can lead to biomechanical alterations of the kinematic chain in which the operated area is involved. There are also some alterations in hip biomechanics after total hip replacement, which are the result of the operation, such as a decrease in walking speed, stride length, the single limb support time and the sagittal plane hip ROM [17]. It is believed that biomechanics that are more natural contribute to better functional results following a surgical treatment. This approach has been proven to be true; although it sometimes forces surgeons to place endoprostheses in positions that are believed to promote faster wear, it does not seem to have adverse effects on implant survival [18]. Because of this, it is important to carefully plan the operation and set the anteversion while considering the “functional safe zone”, hip biomechanics and typical mechanisms of instability and dislocation after THR, such as the impingement of the prosthetic femoral neck on the cup liner; the impingement of the osseous femur on the osseous pelvis; and spontaneous dislocation caused by soft tissue traction without impingement [19].

The numerous papers published in the past year stress the importance of the proper anteversion of the cup [14,16]. Despite this, the current literature provides few examples of studies on cup anteversion outside Lewinnek’s “safe zone” after primary THA. This lack of data about patients’ postoperative outcomes outside this range highlights a shortage of knowledge in this area. The potentially beneficial effect of setting the acetabular anteversion outside the “safe zone” is not achieved when strictly following Lewinnek’s recommendation. Our study is a retrospective study of a small group of patients, designed to evaluate the risk of hip dislocation after setting the acetabular anteversion during THA outside the typical range. Another goal is to highlight the potential value of performing a larger study on patients with an acetabular cup after total hip replacement placed outside the “safe zone”.

## 2. Materials and Methods

### 2.1. Data Design

**Figure 2:**
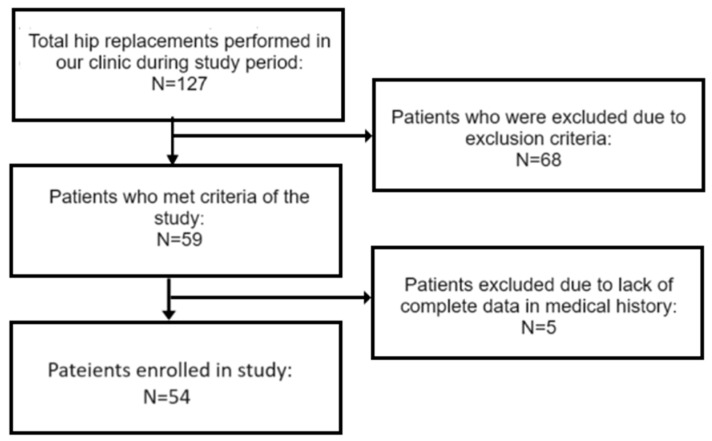
Study population flowchart.

The initial sample size for the study was estimated according to method described by Sim and Lewis [20] and established by general principle to be equal to around 55 patients. Unfortunately, due to differences in regard to the positioning of the implant, being one of the radiological outcome measures for the evaluation of a surgical treatment’s technical effectiveness, it was only possible to gather 10 patients with implants positioned outside Lewinnek’s safe zone within the set period of inquiry. Thus, it was decided to include a sufficient number of patients with implants positioned within Lewinnek’s safe zone and operated on within the same time period as the aforementioned group, to achieve the established sample size and, in this way, to evaluate the possible ratio of patients with implants outside and within the safe zone for future studies. Unfortunately, due to the number of patients excluded from the study in line with the exclusion criteria, N = 54 was the final number of patients included. Hedge’s g was used to estimate the effect size and achieve the power of the tests. The flowchart of the population is in Figure 2.

The inclusion criteria were end-stage arthritis treated by total hip replacement, no backpain and no osteoarthritis of the sacroiliac and vertebral joints in the medical history. The exclusion criteria were

- osteoarthritis of the sacroiliac or vertebral joints in the radiological examination—due to their potential influence on hip joint mobility and pain;
- a previous operation in the area of the hip—to exclude the impacts of previous operations on the hip, including potential restrictions and alterations in the range of motion;
- no follow-up visits—due to a lack of data.

The approval of the ethics committee of the Medical University of Warsaw was received.

### 2.2. Data Collection

**Figure 3:**
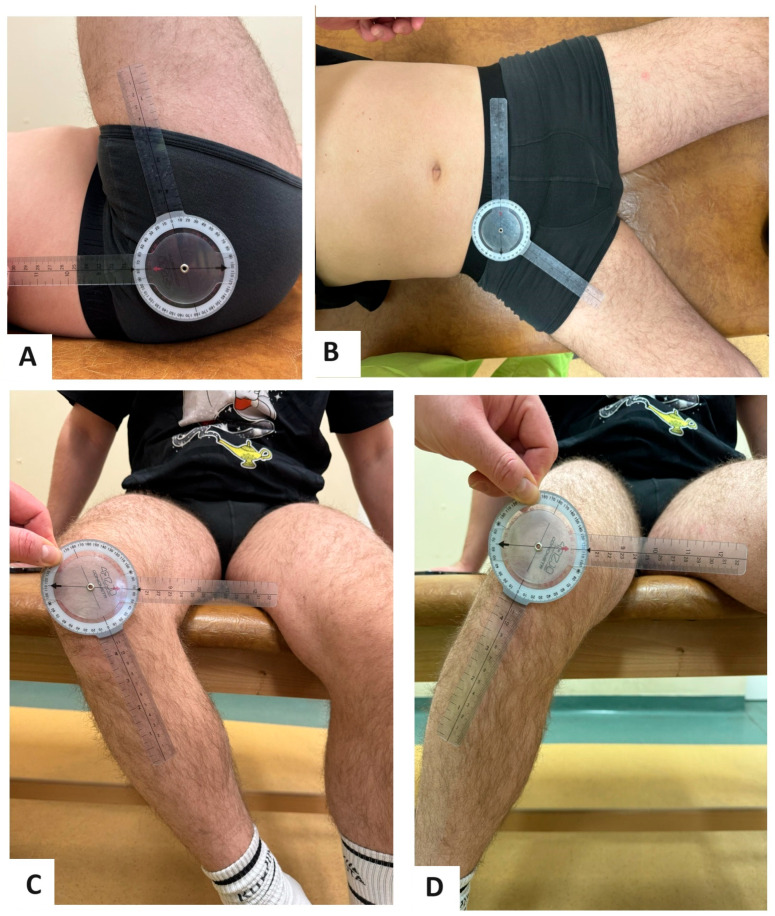
Part of the examination performed preoperatively. The measurement of flexion (**A**), abduction (**B**), external rotation (**C**) and internal rotation (**D**) presented on a model.

Before the operation, patients were prepared in the standard manner with special attention to the physical examination, and the range of movement was measured using a goniometer as showed in Figure 3. The flexion, extension, abduction, adduction, external rotation and internal rotation in flexion were measured.

**Figure 4:**
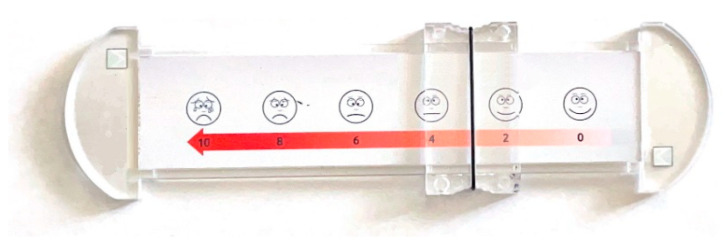
Tool for VAS assessment.

The HHS questionnaire was used to assess the overall functional outcomes before and after the operation. The pain level was assessed on the VAS scale showed in Figure 4. All patients were subjected to THA by a single high-volume surgeon who specialized in arthroplasty (BM). Preoperative planning was performed by a surgeon a day before the planned surgery. The physical examination was performed with special attention paid to the assessment of the spinopelvic mobility and range of movement restrictions.

The procedure was performed on patients positioned laterally, through an anterolateral approach, and the implants used were Taperloc/Fitmore with an Allofit shell (Zimmer Biomet, Warsaw, IN, USA). Radigraphs were taken in the standard manner a day before the operation, a day after the operation, 6 weeks after surgery and 6 months after surgery, during standard follow-up visits. A careful radiological evaluation was performed to exclude any signs of potential instability, including dislocation, malpositioning or loosening of the endoprosthesis components, potential contact between the neck of the prosthesis and the articular component and contact between the bony femur and bony pelvis [19]. The measurement of the anteversion was performed using Lewinnek’s method, which provides excellent reliability in comparison to other methods and CT [21].

**Figure 5:**
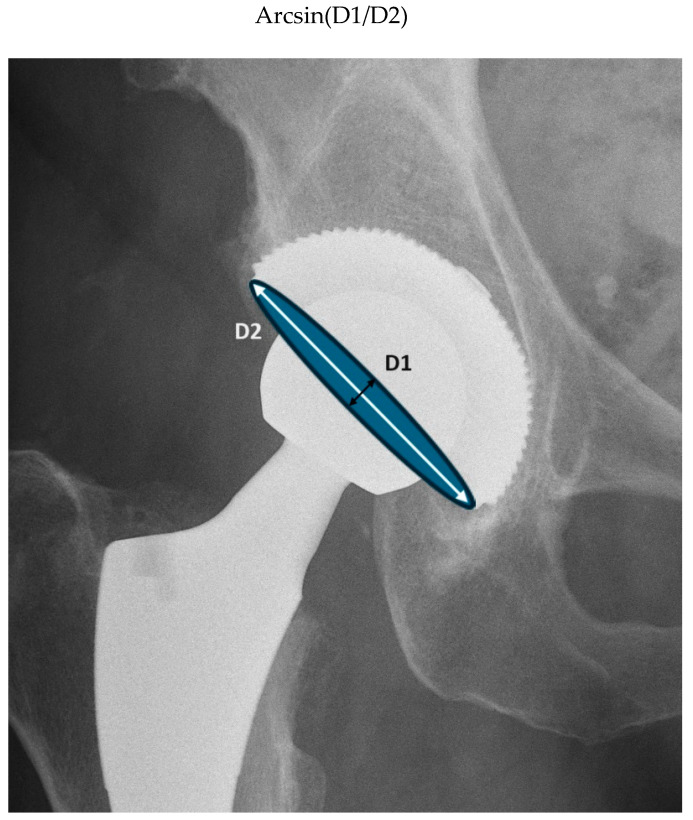
Cup ellipse shadow with two aforementioned diameters: short axis (D1—black arrow) and long axis (D2—white arrow).

**Figure 6:**
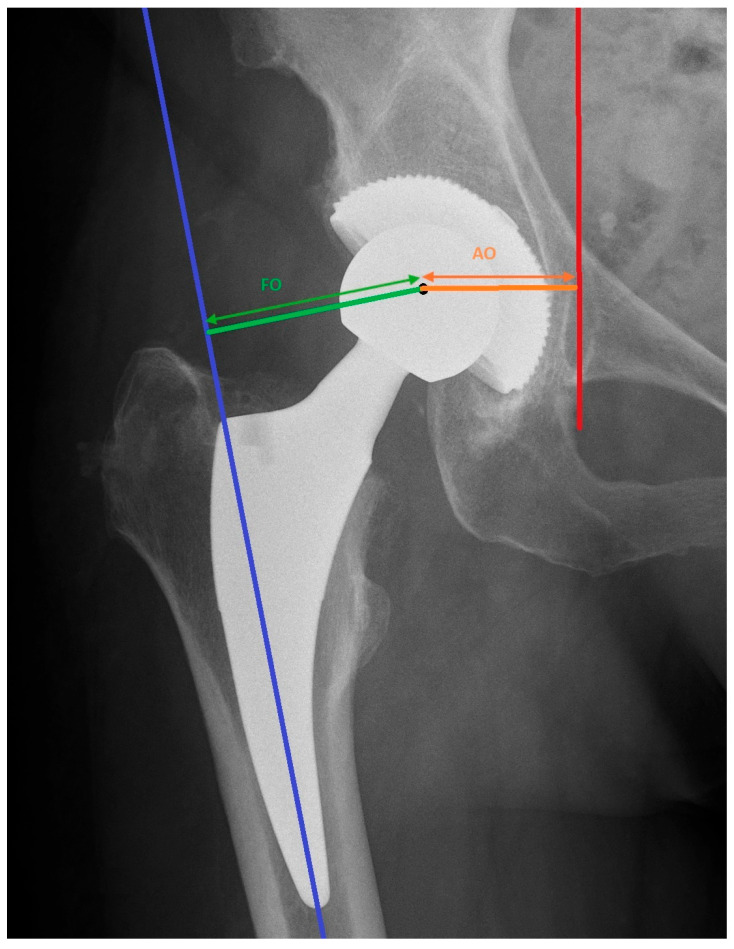
Anatomical axis of the femur (blue line), vertical trans-teardrop line (red line), center of rotation (black point), femoral offset (FO, green arrow), acetabular offset (AO, orange arrow).

To calculate the acetabular anteversion, two measurements were taken: the long axis and short axis of the cup ellipse shadow as presented in Figure 5. Then, the following formula was used:

The offset was measured on standardized radiographs of the pelvis as the sum of two measurements as showed in Figure 6. The first was the distance from the anatomical axis of the femur to the center of rotation in the implant’s head, which is widely known as the femoral offset. Another measurement was performed starting from the center of rotation of the implant to the vertical trans-teardrop line, which is named the acetabular offset. The sum of both measurements was considered as the global offset: FO + AO = GO.

### 2.3. Surgical Technique

The anterolateral approach was applied in the supine position. A longitudinal incision was made in parallel to the femoral axis. The incision started at around 2 cm distal of the greater trochanter and ended at approximately 3–4 cm proximal to the greater trochanter. The exact length of the incision varied and depended on the anatomy of each patient. The subcutaneous tissue was divided and the fascia was located and opened dorsally of the M. tensor fasciae latae. After the blunt dissection of the gluteus medius fascia, an interval between the anterior and medial portions (one third) was developed, and the anterior portion of the muscle was carefully detached. In the next step, the joint was identified, and a ventral capsule resection was performed. After this, the joint was dislocated and the femoral neck visualized and resected according to the preoperative planning. The acetabulum was carefully visualized using retractors and prepared for reaming. In the next step, the acetabulum was reamed down to the planned depth, and the cup was implanted. After this, the leg was rotated externally with adduction to properly visualize the proximal part of the femur. The medullary canal was opened, and the femoral bone was prepared with rasps to gradually reach the planned size. At the end, a trial reduction implant was placed to check the muscle tension, stability and length of the leg. Finally, the trial implant was removed and the determined stem and head were implanted. Part of the gluteus medius was reattached to its insertion. The careful closure of other structures was performed.

### 2.4. Statistical Analysis

A statistical analysis of the results from 54 patients was performed. For comparisons between continuous variables, Student’s *t*-test or Mann–Whitney’s U-test was used, and, for the assessment of the associations between continuous variables, either Pearson’s correlation coefficient or Spearman’s correlation coefficient was used, according to the normality of distribution, examined with the Shapiro–Wilk test. For categorical variables, Fisher’s exact test was performed. For the comparison of the pain levels at defined points in time between the groups, the Aligned Rank Transform was used and a two-way repeated-measures ANOVA with Huynh–Feldt adjustment for within-subject effects was performed. An α value of 0.05 was used to determine the statistical significance of all analyses.

All statistical analyses were conducted using the SAS software, Version 9.4 for Windows (SAS Institute Inc., Cary, NC, USA).

## 3. Results

The analysis was performed on the medical histories of 54 consecutive patients who met the criteria of the study. The patients included 23 males and 31 females. In 23 cases, the operated limb was on the left side; in 31, it was on the right. The mean age of the patients was 69.65 (±9.42) and the mean duration of symptoms was 2.51 (±1.64). Another feature considered vital was the mean BMI, which was 28.53 (±3.61). Patients were examined during regular outpatient visits at 2 weeks, 6 weeks, 3 months and 6 months postoperatively.

Two independent researchers analyzed the medical history preoperatively, from postoperative visits and standardized radiographs, using professional software (Medixant. RadiAnt DICOM Viewer [Software]. Version 2021.1. 27 June 2021).

Patients were divided into two groups according to Lewinnek’s safe zone, which is between 5 and 25 degrees of cup anteversion. The characteristics of the two groups of patients are presented in Table 1, which shows basic features including the age, sex, BMI and duration of symptoms. The interobserver reliability was 0.871, which is considered a high score.

The univariate analysis revealed no significant differences between the group with anteversion within Lewinnek’s safe zone and the group with anteversion outside Lewinnek’s safe zone in regard to the mean HHS score preoperatively, 39.99 (±13.06) vs. 34.67 (±10.03), g = 0.53. power = 0.31, and the mean HHS score postoperatively, 90.75 (±11.54) vs. 91.16 (±12.02), g = 0.04, power = 0.05, as shown in Table 1.

### 3.1. Distribution of Offset and Anteversion

**Figure 7:**
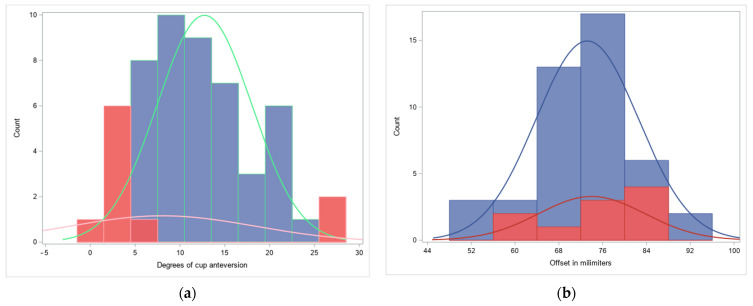
Distribution of postoperative parameters regarding each group: (**a**) cup anteversion; (**b**) total offset. Red color—Group 1, in Lewinnek’s safe zone, blue color—Group 2, outside Lewinnek’s safe zone.

The statistical analysis showed the distribution of the key parameters that were examined in the patients. The distributions of the cup anteversion, measured in degrees, and the offset, which was measured in millimeters, are presented in Figure 7.

### 3.2. Offset Change Regarding Anteversion

In the first step, an analysis was performed to exclude the association between the anteversion and offset change.

**Figure 8:**
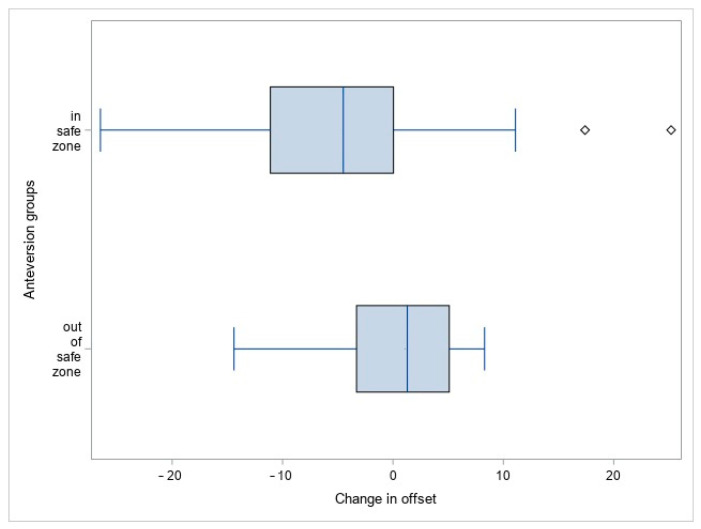
Offset change in two groups of patients (Group 1—in Lewinnek’s safe zone, Group 2—outside Lewinnek’s safe zone, g = 0.58, power = 0.35).

There were no significant differences between the group with anteversion within Lewinnek’s safe zone and the group with anteversion outside Lewinnek’s safe zone in regard to the total offset (78.5 ± 12.4 vs. 74.0 ± 9.7, *p* = 0.8) and change in total offset (−5.3 ± 10.7 vs. 0.56 ± 6.8, *p* = 0.1), as shown in Figure 8.

### 3.3. Anteversion and Postoperative Results in HHS

**Figure 9:**
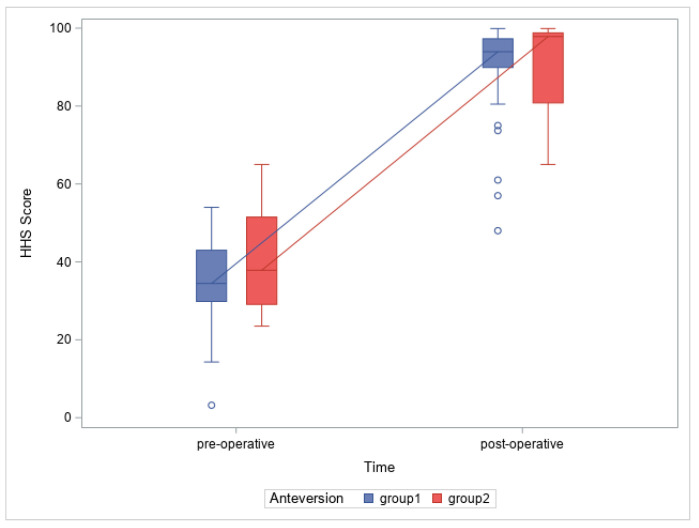
Anteversion and HHS score change (for comparison between preoperative values, g = 0.53, power = 0.31; postoperative values, g = 0.04, power = 0.05; and change in values, g = 0.315, power = 0.14 of HHS, respectively).

There was no statistically significant difference between the groups in regard to the preoperative and postoperative HHS and the change in the scores (*p* = 0.17, *p* = 0.45 and *p* = 0.37, respectively), as presented in Figure 9

### 3.4. Anteversion Impact on Pain

**Figure 10:**
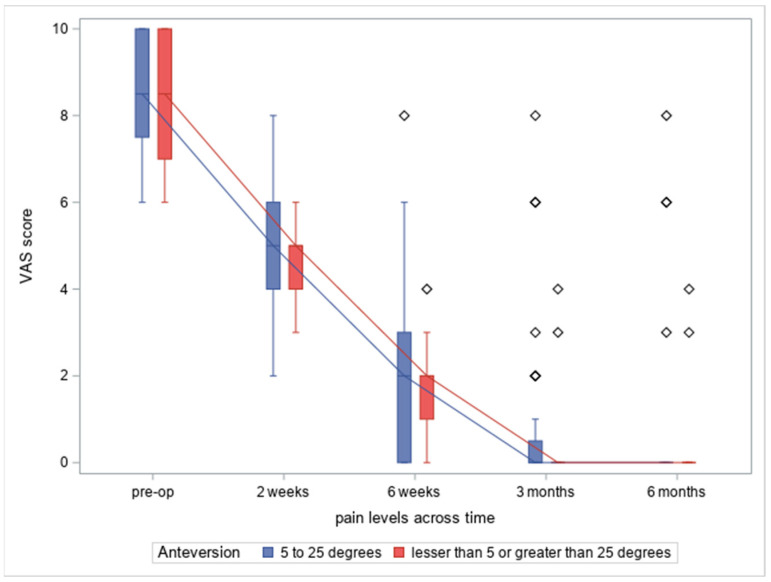
Anteversion and associated pain (VAS scale) at different time points (from the left: before operation, 2 weeks after operation, 6 weeks after operation, 3 months after operation, 6 months after operation).

There was no difference in the pain level variance in the between-subject (*p* = 0.90), within-subject (*p* = 0.96) and factor interaction analyses (*p* = 0.84). No cases of instability or other complications occurred in the two groups. Pain levels presented in Figure 10.

## 4. Discussion

It is well known that there are a number of factors that contribute to the postoperative results in total hip replacement. The latest research shows the importance of a sagittal pelvis imbalance, which could be addressed so as to avoid poor outcomes. It is one of the leading factors contributing to worse functional results and postoperative instability, which may be corrected during operation. Based on these data, it is possible to detect a group of risk factors, including a higher sagittal vertical axis, a higher degree of pelvic incidence minus lumbar lordosis and pelvic retroversion. Another group with a sagittally imbalanced pelvis with anteversion may have a compensatory ability to correct the imbalance after THA and avoid impingements and dislocations [6]. For a few years, our team has devoted efforts to exploring the proper diagnosis of pelvic imbalance in order to optimally plan the operation.

To prepare for an operation on a patient with a sagittal imbalance, the surgeon should always consider alterations in the positioning of the acetabular cup, including inclination and anteversion. Moreover, the usage of high-offset acetabular liners, high-offset femoral stems and dual mobility articulations should be taken into account [22].

First, the factor that is always taken into consideration by our team is the offset. Although it has been investigated, the impact of its alteration is yet not clear. Previously, research showed the best results after THA in patients with restored or decreased offset [23], which could be explained by the lower soft tissue tension and, in consequence, lower pain. After examining the role of the abductor lever arm, it was concluded that we should aim to restore or slightly increase the offset [24]. This statement seems to be true, because proper tension in the muscles around the hip may result in superior functional results. A more recent paper proved that a higher offset may lead to a better ROM [25]. A proper soft tissue balance may not only contribute to ROM improvement but also may prevent, to some extent, dislocation, which corresponds to other findings indicating that a high offset may lead to a lower dislocation rate and greater ROM [26]. Moreover, a study observed a gait improvement in patients with a higher offset [27], favoring the use of a high-offset acetabular liner or high-offset stem.

As mentioned above, variations in offset are generally well tolerated by patients. Our study seems to prove that good results can be obtained regardless of offset changes, but it is important to identify the limitations of this. De Fine et al. show that increasing the offset may not be beneficial, and the usage of modular implants is not indicated [28]. Dual-modular stems were introduced in 1987, and, after initial optimism, a few complications were noted, such as enhanced corrosion in some alloys and mechanical failures. In connection with the fact that there are no clearly stated biomechanical benefits of this implant, its usage is restricted [29]. Other reports clearly show an increased revision rate in dual mobility implants and significantly lower survival, being one of the essential parameters in modern hip alloplasty. Despite the fact that modular stems could be a good option in the case of severe deformities or hip dysplasia, they are rather avoided in primary THA [30]. Our team also support this statement, so the implants used in our research were not modular. In addition to this, lastly, there are studies showing that it is not only a modular implant or greater-offset stem that may increase the offset. We should always take into account the placement of the stem in a slightly varus position [31]. This point of view is supported by other papers in which it is described that a standard stem and short stem provide the same opportunity to restore the offset [32]. Preoperative planning can be made easier by using different neck shaft angle stems, which also may restore the offset well without using modular implants [33].

Another way to address problems of poor outcomes is to use dual mobility implants. As is well known, these implants can offer a solution to recurrent instability, and, due to their properties, they can be used in more difficult cases that need further operative treatment after major complications following primary THA [34]. These implants are not the standard choice for every surgeon, but new research shows that their survival outcomes are acceptable in primary THA, so there is also the possibility to use them to prevent potential complications [35]. Although the advantages of dual mobility implants are unquestionable, orthopedic surgeons must be aware of the potential risks. In recent years, data have shown that dual mobility implants may increase the risk of heterotopic ossification formation [36]. Moreover, researchers show that modular dual mobility implants can increase the risk of adverse reactions to metal debris, which may also contribute to poor postoperative results [37].

The last way to correct a sagittal imbalance is an alteration in the angular position of the cup, including the main factor that was investigated during this research, which is acetabular anteversion. It seems to have a vital role regarding spinopelvic alignment, and the proper planning of this angle can prevent impingement and dislocation [38]. In the late 1970s, a “safe zone” was described by Lewinnek [12] and started to be used as a reference range for many orthopedic surgeons. The latest research shows that this concept is not completely true for every hip [11]. To address this problem, the new term of “functional safe zone” has been introduced, which explains that planning an operation in the standard manner can lead to problems, which could be avoided with a cup position change [38]. According to researchers Lewinnek’s safe zone, in some cases, does not guarantee safety, as it was designed to be applied in a typical hip. In the case of altered spinopelvic alignment or a pelvic imbalance, proper correction should be performed by a hip surgeon, who may be forced to place the cup in a different range from that proposed by Lewinnek. This is the reason for the increased interest in acetabular anteversion and cup placement outside the safe zone [39]. Thus far, there are only few papers taking into account patients outside Lewinnek’s “safe zone” [39,40]. Our research aims to give more insights into this complicated area, highlighting the opportunity to investigate rare series of patient cases with acetabular anteversion outside the safe zone.

For future research, the usage of navigation can also improve the results. In cases where offset restoration is challenging, advanced techniques such as computer-assisted navigation or patient-specific instrumentation can aid in achieving accurate cup positioning. These technologies provide real-time feedback and assist the surgeon in optimizing the implant placement [40], such as intraoperative radiography [41], but we should be cautious when using 2D imaging [42,43].

This study had several limitations. The first was the small group. Since setting the anteversion outside the typical range is quite rare, major problems in the collection of adequate groups occurred. Due to this fact, both patients with higher and lower anteversion were included, with some potential differences in the biomechanical alterations after surgery. Despite this, the group of patients was rather homogeneous, which provided good conditions for the observation of the impact of anteversion alone. This problem may be addressed in future studies by collecting data across a longer period of time, with sample size calculations modified by the results presented in this study, e.g., according to the method described by Sam and Lewis. It may also be possible then to show the potential differences in clinical outcomes between patients with lower or higher values than expected. The second limitation of the study was the restriction to a single surgeon. In this context, the outcomes cannot be generalized and further research is needed. On the other hand, many personal factors related to the surgeon, such as their handedness, skill and experience, etc., were excluded. The third limitation was the use of the angle measurement technique. As the best method for anteversion measurement seems to be computed tomography or MRI, the method applied in this study may be slightly less accurate.

## 5. Conclusions

The role of cup anteversion in total hip replacement is still being examined but seems to be underestimated. In this paper, the authors stressed that it is possible to use a wider range of cup anteversion than was previously widely accepted, without any negative consequences for the postoperative function of the hip. According to results of this study, a mild anteversion change does not seem to change significantly the functional result of the operation, and it does not seem to change patients’ pain postoperatively.

This could help surgeons in planning the operation and shows that, even with some offset changes implemented intraoperatively, the result of the operation can be satisfactory. It may be helpful for surgeons to know the effect of the offset, especially when operating on more difficult cases where offset restoration is not easy or sometimes not possible. Sometimes, struggles to restore the offset impede the results and complicate the operation. The results of this study show that the acceptance of some changes is not correlated with inferior results, especially with regard to function and pain.

Thus far, the impact of an offset change is not fully understood. Although it is not a vital parameter, it is still very important in total hip replacement. Since many findings are inconclusive, further research is needed to fully understand its role.

## Figures and Tables

**Table 1:** Baseline characteristics of enrolled patients divided into two groups.

Feature	In Lewinnek’s Safe Zone N = 44	Outside Lewinnek’s Safe Zone N = 10	*p*-Value
Age (years)	69.6 (±9.82)	69.9 (±7.88)	0.91
BMI	28.55 (±3.78)	28.41 (±2.09)	0.90
Duration of symptoms (years)	2.37 (±1.43)	3.13 (±2.37)	0.19
Inclination	40.33 (±7.02)	37.35 (±5.35)	0.22
Mean HHS score preoperatively	34.67 (±10.03)	39.99 (±13.06)	0.17
Mean HHS score postoperatively	91.16 (±12.02)	90.75 (±11.54)	0.45

## Data Availability

The data that support the findings of this study are available on request from the corresponding author, [P.S.].
